# Early Coronary Angiography Is Associated with Improved 30-Day Outcomes among Patients with Out-of-Hospital Cardiac Arrest

**DOI:** 10.3390/jcm10215191

**Published:** 2021-11-06

**Authors:** Shir Lynn Lim, Yee How Lau, Mark Y. Chan, Terrance Chua, Huay Cheem Tan, David Foo, Zhan Yun Lim, Boon Wah Liew, Nur Shahidah, Desmond R. Mao, Si Oon Cheah, Michael Y. C. Chia, Han Nee Gan, Benjamin S. H. Leong, Yih Yng Ng, Khung Keong Yeo, Marcus E. H. Ong

**Affiliations:** 1 Department of Cardiology, National University Heart Centre, Singapore 119228, Singapore; mark.chan@nus.edu.sg (M.Y.C.); huay_cheem_tan@nuhs.edu.sg (H.C.T.); 2 Department of Cardiology, National Heart Centre, Singapore 169609, Singapore; lau.yee.how@nhcs.com.sg (Y.H.L.); terrance.chua.s.j@singhealth.com.sg (T.C.); yeo.khung.keong@singhealth.com.sg (K.K.Y.); 3 Department of Cardiology, Tan Tock Seng Hospital, Singapore 308433, Singapore; david_foo@ttsh.com.sg; 4 Department of Cardiology, Khoo Teck Puat Hospital, Singapore 768828, Singapore; lim.patrick.zy@ktph.com.sg; 5 Department of Cardiology, Changi General Hospital, Singapore 529889, Singapore; Boon_Wah_Liew@cgh.com.sg; 6 Department of Emergency Medicine, Singapore General Hospital, Singapore 168753, Singapore; nur.shahidah.ahmad@singhealth.com.sg (N.S.); marcus.ong.e.h@singhealth.com.sg (M.E.H.O.); 7 Department of Acute & Emergency Care, Khoo Teck Puat Hospital, Singapore 768828, Singapore; mao.desmond.r@ktph.com.sg; 8 Emergency Medicine Department, Ng Teng Fong General Hospital, Singapore 609606, Singapore; siooncheah@gmail.com; 9 Emergency Department, Tan Tock Seng Hospital, Singapore 308433, Singapore; michael_yc_chia@ttsh.com.sg (M.Y.C.C.); Yih_Yng_NG@ttsh.com.sg (Y.Y.N.); 10 Accident & Emergency, Changi General Hospital, Singapore 529889, Singapore; gan.han.nee@singhealth.com.sg; 11 Emergency Medicine Department, National University Hospital, Singapore 119074, Singapore; benjamin_sh_leong@nuhs.edu.sg; 12 Ministry of Home Affairs, Singapore 329560, Singapore; 13 Health Services and Systems Research, Duke-NUS Medical School, Singapore 169857, Singapore

**Keywords:** coronary angiography, percutaneous coronary intervention, out-of-hospital cardiac arrest, 30-day survival, neurological outcomes

## Abstract

We evaluated the association between early coronary angiography (CAG) and outcomes in resuscitated out-of-hospital cardiac arrest (OHCA) patients, by linking data from the Singapore Pan-Asian Resuscitation Outcomes Study, with a national registry of cardiac procedures. The 30-day survival and neurological outcome were compared between patients undergoing early CAG (within 1-calender day), versus patients not undergoing early CAG. Inverse probability weighted estimates (IPWE) adjusted for non-randomized CAG. Of 976 resuscitated OHCA patients of cardiac etiology between 2011–2015 (mean(SD) age 64(13) years, 73.7% males), 337 (34.5%) underwent early CAG, of whom, 230 (68.2%) underwent PCI. Those who underwent early CAG were significantly younger (60(12) vs. 66(14) years old), healthier (42% vs. 59% with heart disease; 29% vs. 44% with diabetes), more likely males (86% vs. 67%), and presented with shockable rhythms (69% vs. 36%), compared with those who did not. Early CAG with PCI was associated with better survival and neurological outcome (adjusted odds ratio 1.91 and 1.82 respectively), findings robust to IPWE adjustment. The rates of bleeding and stroke were similar. CAG with PCI within 24 h was associated with improved clinical outcomes after OHCA, without increasing complications. Further studies are required to identify the characteristics of patients who would benefit most from this invasive strategy.

## 1. Introduction

Out-of-hospital cardiac arrest (OHCA) is a significant health problem with survival rates of <10% globally [1]. Coronary artery disease (CAD) is one of the leading causes of OHCA [2,3,4], with obstructive CAD found in up to 70% of resuscitated OHCA patients referred for immediate coronary angiography (CAG) [5,6]. Incorporating CAG into the post-resuscitation care bundle may benefit those who do not have an obvious extra-cardiac etiology of OHCA.

Observational studies demonstrate significantly higher survival in comatose OHCA patients undergoing early CAG, regardless of electrocardiogram (ECG) findings [7,8,9]. These studies are limited by selection bias—patients selected for CAG usually have more favorable characteristics and receive more aggressive overall care, which partly accounts for the association with better outcomes. In contrast, the randomized controlled Coronary Angiography after Cardiac Arrest (COACT) trial, Randomized Pilot Clinical Trial of Early Coronary Angiography Versus No Early Coronary Angiography After Cardiac Arrest Without ST-Segment Elevation (PEARL) study, Direct or Subacute Coronary Angiography in Out-of-Hospital Cardiac Arrest (DISCO) pilot study, Angiography after Out-of-Hospital Cardiac Arrest without ST-Segment Elevation (TOMAHAWK) trial, and a recent systematic review failed to show the superiority of early CAG to delayed CAG in improving survival of resuscitated OHCA patients without ST-segment elevation (STE) [10,11,12,13,14]. Current guidelines recommend emergent CAG in OHCA subjects with STE on post-resuscitation ECG, suspected acute myocardial infarction, or evidence of hemodynamic or electrical instability [15]. The extent to which these have been applied to clinical practice is not known. Moreover, quantifying the benefit of early CAG in resuscitated OHCA patients has important implications, particularly in considering the regionalization of post-cardiac arrest care in specialized centers. The purpose of this study was to, using population-based registries, evaluate the association between early CAG and clinical outcomes in resuscitated OHCA patients. We hypothesized early CAG is associated with improved clinical outcomes, mediated by the greater use of revascularization in the early CAG group.

## 2. Materials and Methods

### 2.1. Study Design and Setting

This was a secondary analysis from the Pan-Asian Resuscitation Outcomes Study (PAROS), performed in Singapore, a densely-populated island city-state in Southeast Asia [16]. Data from Singapore were linked with that from the Singapore Cardiac Data Bank (SCDB).

### 2.2. Data Collection

PAROS is an ongoing clinical research network for OHCA in the Asia-Pacific, whose methodology had been previously described [17]. A prospective, multi-center registry, it provides baseline information on OHCA epidemiology, management and outcomes, describes variations among EMS systems, and compares systemic and structural interventions in the Asia-Pacific, with data definitions in accordance with Utstein definitions [18]. Data are extracted from emergency dispatch records, ambulance case notes, and emergency department (ED) and in-hospital records. There are quality assurance data checks built into the data entry system, and data verification checks are implemented to ensure data integrity.

The Singapore Cardiac Data Bank (SCDB) is the main coordinating body to ensure relevant data are collected retrospectively from the public hospitals for the National Medical Audit Meetings (Cardiovascular Discipline), health policy and planning, national disease management, quality assurance, and international benchmarking [19]. Data are obtained from cardiac catheterization, echocardiography and electrocardiogram reports, medical and nursing notes, event logs, and inpatient hospital records. In addition to routine data verification checks, a biennial data quality audit by a third-party auditor is carried out to ensure data quality and integrity.

### 2.3. EMS

The Singapore Civil Defence Force provides nation-wide EMS in Singapore [20]. It is a fire-based system activated by a centralized “995” dispatch system. All ambulances have mechanical cardiopulmonary resuscitation (CPR) devices. As per protocol, all EMS-attended OHCAs are transported to the nearest public hospital. All public hospitals have intensive care units (ICU) capable of instituting targeted temperature management (TTM)—TTM is instituted according to the individual unit’s protocol, and may be surface cooling (Blanketrol II, Cincinnati Sub-Zero, Arctic Sun™, Bard, or Gaymar Meditherm III) or intravascular cooling (ZOLL Thermogard XP^®^) (Table S1). At time of study, five out of six hospitals provided 24-h emergency cardiovascular care.

### 2.4. Study Population and Primary Exposure

We included adult (defined as ≥18 years of age) OHCA patients of presumed cardiac etiology who were successfully resuscitated and eligible for CAG between January 2011 to December 2015. Cardiac arrest was defined as the cessation of cardiac mechanical activity confirmed by the absence of signs of circulation at any time as documented on the EMS treatment record. The etiology of cardiac arrest was identified from the paramedic treatment record, and presumed to be of cardiac cause in the absence of a known cause.

The primary variable of interest was early CAG, defined as CAG performed within 1-calendar day of ROSC. The decision to perform early CAG was a multi-disciplinary process involving an on-call cardiologist and ED physician. A coronary lesion resulting in more than 50% reduction in luminal diameter by visual estimation was considered clinically significant. The presence of thrombus was suggested by the following angiographic features: haziness; reduced contrast density or contrast persistence; irregular lesion contours; or globular filling defects. PCI was attempted if there was an acute coronary occlusion or an unstable lesion considered as the cause of the arrest—this was at the discretion of the interventional cardiologist. Angiographic success was defined as a residual stenosis of <50% with a Thrombolysis In Myocardial Infarction flow grade 3. It was deemed a procedural success if PCI was successfully carried out, and the operator concluded it was a success considering the clinical and procedural events. After the procedures, all patients were admitted to the ICU for further supportive management.

### 2.5. Study Outcomes

The primary outcome was survival status at the 30th day post-arrest (30-day survival). The secondary outcome was the cerebral performance category (CPC) as a measure of functional outcome at the 30th day post-arrest (with CPC 1 or 2, consistent with good neurological outcome).

Data on adverse events, defined as those occurring within 72 h of CAG, were collected for all patients who underwent CAG. Significant bleeding was defined as that resulting in a hemoglobin drop of ≥3 g/dL, requiring transfusion of whole blood or packed red blood cells, or requiring procedural intervention at the bleeding site to stop the bleed. Cardiac tamponade was defined as echocardiographic evidence of pericardial effusion and evidence of systemic hypotension or right heart compromise, requiring intervention. Patients were deemed to have suffered a cerebrovascular accident (CVA) if there was documented loss of neurological function due to ischemia or hemorrhage, with residual symptoms lasting at least 24 h after onset. Finally, we collected data on recurrent ventricular fibrillation (VF) or ventricular tachycardia (VT) necessitating defibrillation.

### 2.6. Statistical Analysis

Continuous variables are presented as mean (standard deviation (SD)) and median (inter-quartile range (IQR))—comparisons were made using an independent sample t-test for parametric data, and a Mann–Whitney U test for non-parametric data. Categorical variables are presented as number (%), and were compared using a chi-squared test. Patients were divided into early CAG and no or delayed CAG groups. Mean imputation was used for missing values for the continuous variable “time from cardiac arrest to first CPR”, and then coded into “time from cardiac arrest to first CPR > 4 min”, after checking for patterns and correlations. Datasets with and without imputation were used in subsequent analyses.

Multivariable logistic regression was performed to identify the predictors associated with 30-day survival and favorable neurological outcome (CPC 1 or 2). Aside from early CAG, the factors considered included age, sex, race, history of heart disease, location of arrest, witnessed OHCA, initial cardiac arrest rhythm, pre-hospital defibrillation, bystander cardiopulmonary resuscitation, time from cardiac arrest to first CPR > 4 min, and the use of epinephrine. The Box–Tidwell test was used to prove the linearity between logit of outcomes and continuous variables. A subsequent multivariable logistic regression with a categorical variable defined by no or delayed CAG, early CAG without early PCI, and early CAG with PCI was performed to explore the effects of revascularization. Based on intention-to-treat principles, the patients were classified to have received PCI regardless of procedural success.

To address the confounding by indication, propensity score adjustment was performed to model the actual treatment decision. Inverse probability weighted estimator (IPWE), a propensity score adjustment method which maintains sample size and preserves external validity, was applied to assess the robustness of the results from the multivariable logistic regression. Propensity scores were first estimated using binary logistic regression, based on factors mentioned above, with early CAG as an outcome (Table S2). A regression model based on generalized estimating equation, adjusted for a weight equal to the inverse of the propensity score for the treatment group and 1-inverse of the propensity score for the control group, was applied.

*p*-value of < 0.05 was considered statistically significant, and odds ratios were derived with 95% confidence intervals. Covariate balance of IPWE was assessed using the “WeightIt” and “cobalt” packages of R programming (The R Foundation for Statistical Computing, Vienna, Austria). Statistical analysis was performed using the statistical package IBM SPSS Statistics Version 26.0.0.0 (IBM Corp, Armonk, NY, USA), and R programming version 3.6.3.

## 3. Results

### 3.1. Demographics, Event Characteristics and Outcomes

**Figure 1:**
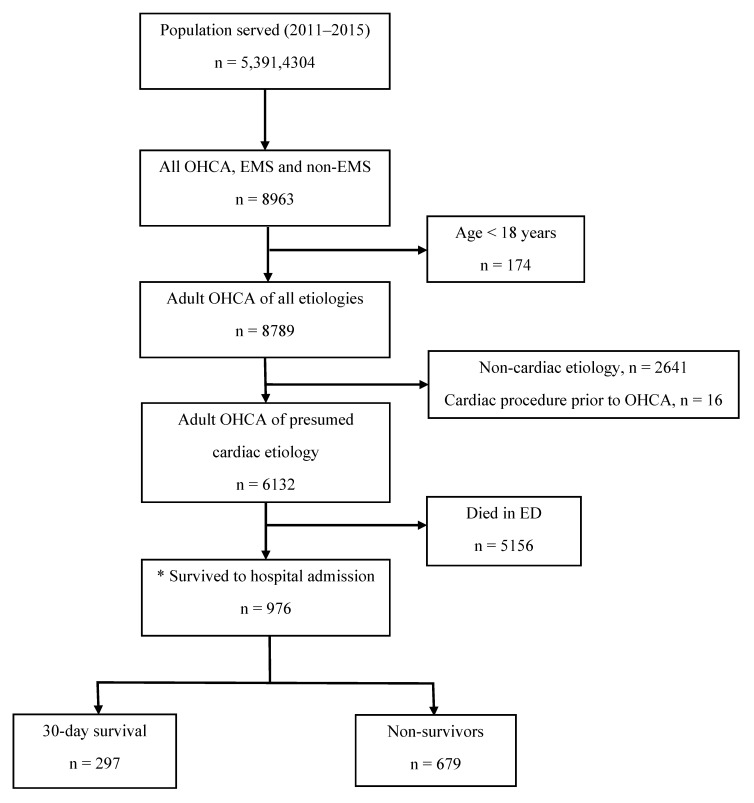
Patient selection. * Refers to the study population. Abbreviations: OHCA, out-of-hospital cardiac arrest; EMS, Emergency Medical Services; ED, Emergency Department.

From 1 January 2011 to 31 December 2015, 976 consecutive OHCA patients of presumed cardiac etiology survived to admission, and were included in the analysis (Figure 1). Their characteristics are summarized in Table 1. Patients who underwent early CAG were younger, more likely male, and had fewer co-morbidities, compared with those with no or delayed CAG. The proportion who presented with an initial shockable rhythm and achieved pre-hospital ROSC amongst those who underwent early CAG was almost doubled that of those who did not. There was an increasing trend in the use of early CAG over the years (*p* = 0.037 for trend).

### 3.2. Prevalence of CAD

Characteristics of CAD, treatment, and procedural complications are summarized in Table 2. The majority of patients who underwent CAG did so within 1-calendar day. Obstructive CAD was highly prevalent, and thrombus was reported in one-third of the patients, irrespective of timing of CAG. Triple vessel disease (TVD) was more prevalent in the early CAG group. Two-thirds of CAG patients underwent revascularization, the majority via PCI. Patients who underwent PCI had a lower prevalence of heart disease, but presented more with an initial shockable rhythm (Table S3). The use of coronary artery bypass graft surgery (CABG) was similar in both groups. The use of prasugrel or ticagrelor was two times higher in the early CAG group. VT or VF requiring cardioversion was almost three times higher in the early CAG group—there were no appreciable differences in the rates of bleeding, CVA, or cardiac tamponade.

### 3.3. Survival and Neurological Outcomes

**Figure 2:**
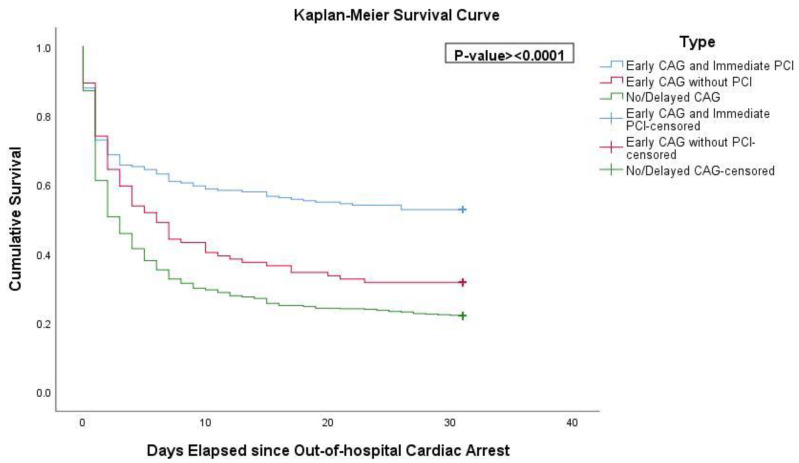
Kaplan–Meier survival curve. Abbreviations: CAG, coronary angiography; PCI, percutaneous coronary intervention.

Resuscitated OHCA patients who underwent early CAG showed significantly higher rates of 30-day survival and good neurological outcome compared with patients with no or delayed CAG. In particular, the unadjusted 30-day survival was more than two-fold higher in patients who underwent early CAG with PCI, compared with no or delayed CAG (Figure 2).

Most of the predictors considered were significant in analyzing survival and good neurological outcome. Race and pre-hospital defibrillation were non-significant in the univariate analyses, and removed from the final models.

**Figure 3:**
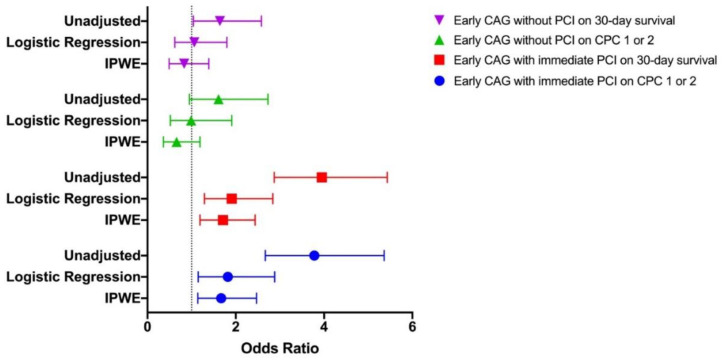
Associations between early CAG, with and without immediate PCI, and outcomes. No or delayed CAG was taken as the reference group. Abbreviations: CAG, coronary angiography; PCI, percutaneous coronary intervention; IPWE, inverse probability weighted estimator; CPC, cerebral performance category.

The subsequent analyses with logistic regression found some of the predictors were significant in explaining the outcomes (Table 3a). The odds of survival significantly increased with an initial shockable rhythm and early CAG, but decreased with age, history of heart disease, arrest at home, and epinephrine administration. The odds of good neurological outcome similarly increased with initial shockable rhythm, bystander CPR, and early CAG, but decreased with age, arrest at home, time from cardiac arrest to first CPR > 4 min, and epinephrine administration. Subsequent analysis showed early CAG increased the odds of survival and good neurological outcome only when coupled with immediate PCI (Table 3b and Figure 3). Clinically relevant two-way interaction terms were evaluated, but none were statistically significant.

IPWE adjustment was carried out using the same set of predictors which were significant in predicting outcomes in the univariable analyses, and available to physicians before triage into early versus no or delayed CAG. Compared with no or delayed CAG, early CAG did not significantly increase the odds of survival or good neurological outcome (Table S4a and Figure 3). Subsequent analyses accounting for revascularization showed the odds of survival and good neurological outcome significantly increased when early CAG was coupled with immediate PCI (Table S4b,c and Figure 3).

The distributional balance plots for propensity score show that there was an improvement in the balance between early CAG and no or delay CAG groups after IPWE (Figure S1). Most of the covariates display a standardized mean difference of below 0.1 and 0.25 after IPWE, which are the general thresholds for covariate imbalance (Figure S2).

## 4. Discussion

In this nationwide study involving adult OHCA of presumed cardiac etiology, we observed a significant association between early CAG with improved survival and neurological outcome, driven by early revascularization. This association was robust to adjustment for propensity to perform the procedure, which accounted for the more favorable characteristics of the patients who received early CAG. Rates of complications did not differ significantly between the two groups.

### 4.1. Comparison of Findings with Prior Studies

The majority of OHCA in Singapore are cardiac in origin, with the 69.8% in our study consistent with what is reported worldwide [2,3,4]. The rationale for benefit of early CAG and revascularization lies in the notion of acute coronary syndromes (ACS) driving the majority of OHCA, especially in cases with no obvious extra-cardiac cause. In these patients, plaque rupture and disruption of flow in the culprit coronary artery ostensibly led to electrical and hemodynamic instability. Early revascularization, through restoring the coronary circulation, may benefit by preventing recurrent cardiac instability.

The evidence supporting a benefit of early CAG in OHCA comes entirely from observational studies with a heterogenous magnitude of effect, in part, related to the variations in sample size, patient characteristics, and methodological rigor [7,8,9]. In particular, few studies accounted for confounding by indication, which may have led to an over-estimation of the benefit of early CAG in such studies. Our study, which adjusted for propensity to perform the procedure, found early CAG with immediate PCI increased the odds of survival and good neurological recovery by almost two-fold, with minimal impact on bleeding risks, findings that corroborated with recent propensity-adjusted studies [21,22,23,24]. Collectively, these results imply that revascularization with PCI drives the benefit of early CAG. Yet, they run contrary to the randomized controlled COACT, PEARL, and TOMAHAWK trials [10,12,14], which may be explained by the following: (1) We included all resuscitated OHCA patients of presumed cardiac etiology, regardless of ECG findings. It is conceivable that a significant proportion of the benefits of early CAG and revascularization seen in our study were driven by those with STEMI. Unfortunately, post-resuscitation ECGs were not available for this study. (2) Despite differences in inclusion criteria of COACT, PEARL, and TOMAHAWK, these studies uniformly saw less than 40% of study population undergoing coronary interventions, in contrast to 70% of our CAG population, implying only a small proportion of subjects in those studies would be affected by the performance of CAG. (3) Our definition of early CAG, though similar to that used in observational studies, [22,23] differed from those used in RCTs, which adopted an immediate CAG approach. We assumed CAG performed within 1-calendar day to be immediate based on knowledge of existing practices, but it remains plausible that some of these early CAG were performed after transfer to the ICU when patients demonstrated clear evidence of myocardial ischemia. (4) Differences in post-resuscitation care between our study population and those enrolled in RCTs may have altered the risk-benefit ratio of early CAG and PCI. Only 26% of our patients received TTM, in contrast to more than 75% of the study population of COACT, PEARL, and TOMAHAWK—this could be due to a combination of our hospitals being in the early adoption phase of TTM during the study period, and the use of protocols with strict inclusion criteria. (5) Neurological injury was the top cause of mortality in all three trials, for which early cardiac interventions would have little influence over. The ACCESS to the Cardiac Cath Lab in Patients Without STEMI Resuscitated from Out-of-hospital VT/VF Cardiac Arrest RCT (ClinicalTrials.gov Identifier: NCT03119571) was unfortunately terminated in January 2021 due to low enrolment. The ongoing Direct of Subacute Coronary Angiography in Out-of-hospital Cardiac Arrest—a Prospective, Randomized Study (ClinicalTrials.gov Identifier: NCT02309151) may help address this important question.

### 4.2. Practice of Early CAG in Singapore

Our study reported a lower prevalence of early CAG and PCI, compared with other studies with similar populations after OHCA [22,23]. The low prevalence rates may be related to perceived futility amongst managing physicians locally, compounded by the implications of increased post-procedure mortality and complications. Nevertheless, we noticed an upward trend in the use of early CAG over the years. A significant number of studies, with relatively modest sample sizes, published between 2000 and 2015, have found improved outcomes with early CAG after OHCA [5,21,23,25,26,27,28]. These led to major cardiovascular professional societies issuing recommendations on the use of coronary angiography in OHCA from 2006 onwards, and most likely influenced the increasing use of CAG after OHCA in our study [29,30,31].

### 4.3. The Use of CABG as a Revascularization Strategy

The use of CABG in our study was comparable between both groups, despite a higher prevalence of TVD in the early CAG group. The relatively lower use of CABG in the early CAG group may be due to managing physicians’ perceived risk-benefit ratio of this major operation. Firstly, subjecting these critically ill patients to early CABG ran the risk of additional inflammation, trauma, and increased risk of stroke. More importantly, there was uncertainty over the neurological recovery of these patients. The strategy of deferring CABG until after neurological recovery achieved was adopted by the COACT trial [10], underscoring the reservations towards early CABG in this group of patients.

### 4.4. Adverse Events

Higher rates of bleeding, clinically significant or not, have been reported with early CAG in OHCA [22,32]. Our study showed neither increased rates of significant bleeding nor other procedural-related complications, including cardiac tamponade and cerebrovascular accident. There were increased rates of ventricular tachycardia and ventricular fibrillation amongst those who underwent early CAG, reflecting a group that was clinically unstable. Knowledge of the rates of acute kidney injury would have been good to have—unfortunately, these data were not captured by our databases.

### 4.5. Clinical Implications

Early CAG and revascularization should be viewed in the larger context of post-resuscitation care, and identifying the patients who would benefit the most is key: high-risk ACS patients with unstable coronary lesions, and the potential for neurological recovery following successful revascularization. The identification of these high-risk ACS patients is challenging, partly because of the lack of clinical signs of ischemic and limited diagnostic utility of initial shockable rhythm, troponin levels, ECG changes other than ST-segment elevation, and echocardiographic abnormalities. Furthermore, the timing of post-ROSC ECG may influence its diagnostic accuracy [33]. Factors associated with PCI in our study, an initial shockable rhythm and the absence of pre-existing heart disease, are insufficient as selection criteria. A systematic coronary angiogram in all resuscitated OHCA patients with no obvious extra-cardiac etiology may not be cost-effective, given neurological injury is the top cause of mortality in observational and randomized controlled studies. The likelihood of neurologic recovery cannot be determined reliably at the time emergency cardiovascular interventions are being carried out. There is a need to improve prediction of unstable coronary lesions and early neuroprognostication, in order to identify patients who would benefit the most from early CAG and revascularization.

### 4.6. Strengths and Limitations

The strengths of our study include the population-based design of registries with uniform data collection based on Utstein definitions for reporting cardiac arrest and the capture of all EMS-attended OHCA cases. Both registries have in-built quality control measures, and regular data audits therefore ensure data quality and integrity. We adjusted for propensity to perform CAG, thereby reducing confounding by indication. Our study should be interpreted in the context of the following limitations. The observational nature of the study precluded causality. There was clear selection bias favouring subjects undergoing early CAG—some bias likely remained despite attempts to correct this statistically. We included patients admitted from 2011 to 2015, and could not discount changes in patient profile and management protocols, which may alter the clinical applicability of our findings. These include the increasing incidence of OHCA and proportion of older patients (>65 years old), improvements in pre-hospital care in Singapore, and the increased use of TTM, all of which may alter the risk-benefit ratio of early CAG and PCI. We lacked information on socioeconomic factors, hospital-based management, cause of death, and long-term functional outcomes. Importantly, we lacked data on post-resuscitation ECGs, which precluded our ability to distinguish ST-segment myocardial infarction (STEMI) versus non-STEMI—we also lacked data on rates of stent thrombosis and re-intervention. Both databases were early in their development and collected mainly essential pre-hospital and coronary procedural data variables. The institutions had not fully adopted electronic medical records, and ECGs were not digitized, thereby preventing us from retrospectively collecting these data. There were varying amounts of missing data for all OHCA cases, albeit a small proportion (<2%). Finally, as with all epidemiological studies, data integrity, validity, ascertainment bias, and misclassifications were potential limitations.

## 5. Conclusions

CAG with PCI within 24 h was associated with improved clinical outcomes after OHCA, without increasing complications. Further studies are required to refine the selection criteria to identify those patients who would benefit most from this invasive strategy.

## Figures and Tables

**Table 1:** Characteristics of resuscitated adult OHCA patients of presumed cardiac etiology.

	Early CAG (*n* = 337)	No/Delayed CAG (*n* = 639)	*p* Value *
Demographics			
Mean age (yrs), mean (SD)	60.2 (12.1)	65.8 (13.5)	<0.001
Male sex, *n* (%)	290 (86.1)	429 (67.1)	<0.001
Ethnicity - Chinese, *n* (%) - Malay, *n* (%) - Indian, *n* (%) - Others, *n*	209 (62.0) 44 (13.1) 57 (16.9) 27 (8.0)	420 (65.7) 108 (16.9) 85 (13.3) 26 (4.1)	0.013
Medical History			
Hypertension, *n* (%)	183 (54.3)	401 (62.8)	0.013
Diabetes mellitus, *n* (%)	99 (29.4)	280 (43.8)	<0.001
Hyperlipidemia, *n* (%)	136 (40.4)	306 (47.9)	0.029
Heart disease, *n* (%)	140 (41.5)	377 (59.0)	<0.001
Renal disease, *n* (%)	33 (9.8)	142 (22.2)	<0.001
Respiratory disease, *n* (%)	19 (5.6)	88 (13.8)	<0.001
Stroke, *n* (%)	20 (5.9)	89 (13.9)	<0.001
Event information			
Arrest at home residence, *n* (%)	150 (44.5)	419 (65.6)	<0.001
Witnessed arrest, *n* (%)	273 (81.0)	477 (74.6)	0.031
Initial shockable rhythm, *n* (%) **	234 (69.4)	227 (35.5)	<0.001
Pre-hospital interventions			
Bystander CPR, *n* (%)	169 (50.1)	281 (44.0)	0.076
Bystander AED, *n* (%)	17 (5.0)	23 (3.6)	0.812
Pre-hospital ROSC, *n* (%)	140 (41.5)	149 (23.3)	<0.001
Timings			
EMS response time (min), median (IQR)	7.7 (6.2,9.8)	8.0 (6.4,10.1)	0.108
No-flow time (min), median (IQR)	11.8 (6.0,16.0)	12.7 (8.0,18.0)	0.005
Low-flow time (min), median (IQR)	7.6 (3.3,16.1)	9.7 (5.8,17.8)	0.069
CA to CPR > 4 min, *n*(%) ***	254 (79.9)	516 (85.3)	0.045
CPR to ROSC > 20 min, *n*(%) ****	16 (12.7)	24 (19.4)	0.207
Hospital interventions			
Epinephrine administration, *n* (%)	180 (53.4)	323 (50.5)	0.433
ECMO, *n* (%)	4 (1.2)	2 (0.3)	0.219
TTM, *n* (%)	121 (35.9)	137 (21.4)	<0.001
Outcomes			
30-day survival, *n* (%)	156 (46.3)	141 (22.1)	<0.001
Survival with CPC 1 or 2, *n* (%)	108 (32.0)	87 (13.6)	<0.001

Numbers are *n* (%) for categorical variables and median (interquartile range (IQR)) for continuous variables, except for age, which is expressed in mean (SD). *p*-value is for differences between early CAG versus no/delayed CAG by X_2_ test for categorical variables, and Mann–Whitney U test for continuous variables. Yate’s continuity correction is applied for 2 × 2 tables. Details on medical history are obtained from hospital electronic medical records. Heart disease is defined as the presence of any documented coronary artery disease or cardiomyopathy. Renal disease is defined as the presence of any documented chronic kidney disease, and this includes end-stage renal disease on renal replacement therapy. TTM was administered using intravascular cooling in 6 (1.8%) of patients undergoing early CAG, and 12 (1.9%) patients receiving no or delayed CAG. Abbreviations: CAG, coronary angiography; CPR, cardiopulmonary resuscitation; ROSC, return of spontaneous circulation; EMS, emergency medical services; CA to CPR, time from cardiac arrest to first CPR; CPR to ROSC, time from first CPR to ROSC; ECMO, extra-corporeal membrane oxygenation; TTM, targeted temperature management; CPC, Cerebral Performance Category. * Statistically significant at 5%. ** Initial shockable rhythm refers to first arrest rhythm which was ventricular fibrillation, ventricular tachycardia, or unknown shockable rhythm. *** Counts for early CAG (*n* = 318) and no/delayed CAG (*n* = 605). **** Counts for early CAG (*n* = 126) and no/delayed CAG (*n* = 124).

**Table 2:** Characteristics of coronary artery disease, treatment, and complications.

	Early CAG (*n* = 337)	Delayed CAG (*n* = 64)	*p*-Value *
Severity of coronary artery disease - Normal/minor CAD - Single vessel disease - Double vessel disease - Triple vessel disease	36 (10.7) 88 (26.1) 66 (19.6) 147 (43.6)	13 (20.3) 17 (26.6) 15 (23.4) 19 (29.7)	0.072
Presence of thrombus **	59(32.8%)	10(31.3%)	1.000
Revascularization treatment - PCI - CABG (includes those with PCI) - Medical therapy	230 (68.2) 8 (2.4) 99 (29.4)	32 (50.0) 2 (3.1) 30 (46.9)	0.018
Antiplatelet therapy *** - Aspirin - Clopidogrel - Ticagrelor/Prasugrel	175 (83.3) 100 (47.6) 72 (34.3)	31 (93.9) 21 (63.6) 5 (15.2)	0.188 0.128 0.046
^∆^Procedural success	214 (91.8)	28 (87.5)	0.628
IABP ****	48 (14.2)	5 (7.8)	0.234
ECMO ****	12 (3.6)	2 (3.1)	1.000
Complications ** - Bleeding (GI/GU) - VF/VT requiring cardioversion - Stroke - Tamponade	5 (2.0) 42 (17.1) 4 (1.6) 0 (0.0)	0 (0.0) 3 (5.8) 1 (1.9) 0 (0.0)	0.300 0.039 0.880 NA

Numbers are *n* (%) for categorical variables. *p*-value is for differences between early CAG versus no/delayed CAG by X_2_ test for categorical variables. Yate’s continuity correction is applied for 2 × 2 tables. Abbreviations: CAG, coronary angiography; CAD, coronary artery disease; PCI, percutaneous coronary intervention; CABG, coronary artery bypass graft surgery; IABP, intra-aortic balloon pump; ECMO, extra-corporeal membrane oxygenation; GI, gastrointestinal; GU, genitourinary; VF, ventricular fibrillation; VT, ventricular tachycardia. ^∆^Relevant for PCI only. Counts for early CAG(*n* = 233) and delayed CAG(*n* = 32). * Statistically significant at 5%. ** Data were available only from the year 2013 onwards. Counts for early CAG(*n* = 180) and delayed CAG(*n* = 32). *** Includes those administered pre/post CAG. Counts for early CAG (*n* = 210) and delayed CAG (*n* = 33). **** Includes those done pre/post CAG.

**Table 3:** Predictors of outcomes by logistic regression.

(a): Early CAG regardless of revascularization.				
	**30-Day Survival**		**Discharged with CPC 1 or 2**	
**Variable**	**Adjusted OR (95% CI)**	***p*-Value**	**Adjusted OR (95% CI)**	***p*-Value**
Age	0.98 (0.97,0.99)	0.001	0.96 (0.95,0.98)	<0.001
Male sex	1.01 (0.67,1.52)	0.956	1.37 (0.82,2.29)	0.230
Heart disease	0.60 (0.42,0.84)	0.004	0.80 (0.53,1.22)	0.307
Arrest at home	0.52 (0.37,0.73)	<0.001	0.46 (0.31,0.69)	<0.001
Witnessed arrest	1.39 (0.90,2.16)	0.139	1.35 (0.77,2.38)	0.296
Initial shockable rhythm	5.72 (3.96,8.26)	<0.001	6.24 (3.88,10.04)	<0.001
Bystander CPR	1.19 (0.83,1.71)	0.345	1.69 (1.08,2.64)	0.021
CA to CPR >4 min	0.72 (0.43,1.18)	0.190	0.36 (0.20,0.64)	0.001
Epinephrine administration	0.29 (0.21,0.41)	<0.001	0.14 (0.09,0.22)	<0.001
Early CAG	1.59 (1.12,2.26)	0.010	1.54 (1.01,2.34)	0.045
(b) Early CAG with and without immediate PCI.				
	**30-Day Survival**		**Discharged with CPC 1 or 2**	
**Variable**	**Adjusted or (95% CI)**	***p*-Value**	**Adjusted or (95% CI)**	***p*-Value**
Age	0.98 (0.97,0.99)	0.001	0.96 (0.95,0.98)	<0.001
Male sex	1.00 (0.67,1.51)	0.984	1.35 (0.81,2.27)	0.255
Heart disease	0.63 (0.44,0.89)	0.010	0.85 (0.55,1.30)	0.451
Arrest at home	0.53 (0.38,0.74)	<0.001	0.46 (0.31,0.70)	<0.001
Witnessed arrest	1.38 (0.89,2.13)	0.155	1.33 (0.76,2.35)	0.320
Initial shockable rhythm	5.64 (3.90,8.16)	<0.001	6.17 (3.83,9.95)	<0.001
Bystander CPR	1.19 (0.83,1.71)	0.337	1.72 (1.10,2.69)	0.018
CA to CPR >4 min	0.73 (0.44,1.22)	0.229	0.37 (0.20,0.66)	0.001
Epinephrine administration	0.29 (0.21,0.41)	<0.001	0.14 (0.09,0.22)	<0.001
Coronary interventions				
-No or delayed CAG	Reference	Reference		
-Early CAG without PCI	1.06 (0.62,1.80)	0.843	0.99 (0.52,1.91)	0.984
-Early CAG with immediate PCI	1.91 (1.29,2.84)	0.001	1.82 (1.15,2.88)	0.010

Predictors of outcomes based on logistic regression using the categorical variable defined by (a) no or delayed CAG (reference) versus early CAG, and (b) no or early CAG (reference) versus early CAG without PCI versus early CAG with immediate PCI. Results were based on analyses performed using imputed dataset. Findings were similar to (a) without imputation, and (b) inclusion of pre-hospital defibrillation, which was not significant in univariate analyses. Abbreviations: CAG, coronary angiography; CPC, Cerebral Performance Category; OR, odds ratio; CI, confidence interval; CPR, cardiopulmonary resuscitation; CA to CPR, time from cardiac arrest to first CPR; CAG, coronary angiography; PCI, percutaneous coronary intervention.

## Data Availability

Data supporting the findings of this study are available from the corresponding author upon reasonable request, subject to approval by the local institutions.

## Supplementary Materials

The following are available online at https://www.mdpi.com/article/10.3390/jcm10215191/s1, Figure S1: Distributional balance for propensity score, Figure S2, Covariate balance before and after IPWE, Table S1: Summary of TTM practices according to institution, Table S2: Logistic regression of predictors for early CAG, Table S3: Characteristics of resuscitated adult OHCA patients who received CAG, Table S4: Association between early CAG with outcomes.

## References

[1] Berdowski J., Berg R.A., Tijssen J.G., Koster R.W. (2010). Global incidences of out-of-hospital cardiac arrest and survival rates: Systematic review of 67 prospective studies. Resuscitation.

[2] Virani S.S., Alonso A., Benjamin E.J., Bittencourt M.S., Callaway C.W., Carson A.P., Chamberlain A.M., Chang A.R., Cheng S., Delling F.N. (2020). Heart Disease and Stroke Statistics-2020 Update: A Report from the American Heart Association. Circulation.

[3] Beck B., Bray J., Cameron P., Smith K., Walker T., Grantham H., Hein C., Thorrowgood M., Smith A., Inoue M. (2018). Regional variation in the characteristics, incidence and outcomes of out-of-hospital cardiac arrest in Australia and New Zealand: Results from the Aus-ROC Epistry. Resuscitation.

[4] Gräsner J.T., Lefering R., Koster R.W., Masterson S., Böttiger B.W., Herlitz J., Wnent J., Tjelmeland I.B., Ortiz F.R., Maurer H. (2016). EuReCa ONE-27 Nations, ONE Europe, ONE Registry: A prospective one month analysis of out-of-hospital cardiac arrest outcomes in 27 countries in Europe. Resuscitation.

[5] Dumas F., Cariou A., Manzo-Silberman S., Grimaldi D., Vivien B., Rosencher J., Empana J.P., Carli P., Mira J.P., Jouven X. (2010). Immediate percutaneous coronary intervention is associated with better survival after out-of-hospital cardiac arrest: Insights from the PROCAT (Parisian Region Out of hospital Cardiac ArresT) registry. Circ. Cardiovasc. Interv..

[6] Spaulding C.M., Joly L.M., Rosenberg A., Monchi M., Weber S.N., Dhainaut J.F., Carli P. (1997). Immediate coronary angiography in survivors of out-of-hospital cardiac arrest. N. Engl. J. Med..

[7] Khan M.S., Shah S., Mubashir A., Khan A.R., Fatima K., Schenone A.L., Khosa F., Samady H., Menon V. (2017). Early coronary angiography in patients resuscitated from out of hospital cardiac arrest without ST-segment elevation: A systematic review and meta-analysis. Resuscitation.

[8] Khera R., CarlLee S., Blevins A., Schweizer M., Girotra S. (2018). Early coronary angiography and survival after out-of-hospital cardiac arrest: A systematic review and meta-analysis. Open Heart.

[9] Camuglia A.C., Randhawa V.K., Lavi S., Walters D.L. (2014). Cardiac catheterization is associated with superior outcomes for survivors of out of hospital cardiac arrest: Review and meta-analysis. Resuscitation.

[10] Lemkes J.S., Janssens G.N., van der Hoeven N.W., Jewbali L., Dubois E.A., Meuwissen M., Rijpstra T.A., Bosker H.A., Blans M.J., Bleeker G.B. (2019). Coronary Angiography after Cardiac Arrest without ST-Segment Elevation. N. Engl. J. Med..

[11] Verma B.R., Sharma V., Shekhar S., Kaur M., Khubber S., Bansal A., Singh J., Ahuja K.R., Nazir S., Chetrit M. (2020). Coronary Angiography in Patients with out-of-Hospital Cardiac Arrest without ST-Segment Elevation. JACC Cardiovasc. Interv..

[12] Kern K.B., Radsel P., Jentzer J.C., Seder D.B., Lee K.S., Lotun K., Janardhanan R., Stub D., Hsu C.H., Noc M. (2020). Randomized Pilot Clinical Trial of Early Coronary Angiography Versus No Early Coronary Angiography after Cardiac Arrest without ST-Segment Elevation: The PEARL Study. Circulation.

[13] Elfwén L., Lagedal R., Nordberg P., James S., Oldgren J., Böhm F., Lundgren P., Rylander C., van der Linden J., Hollenberg J. (2019). Direct or subacute coronary angiography in out-of-hospital cardiac arrest (DISCO)-An initial pilot-study of a randomized clinical trial. Resuscitation.

[14] Desch S., Freund A., Akin I., Behnes M., Preusch M.R., Zelniker T.A., Skurk C., Landmesser U., Graf T., Eitel I. (2021). Angiography after Out-of-Hospital Cardiac Arrest without ST-Segment Elevation. N. Engl. J. Med..

[15] Callaway C.W., Donnino M.W., Fink E.L., Geocadin R.G., Golan E., Kern K.B., Leary M., Meurer W.J., Peberdy M.A., Thompson T.M. (2015). Part 8: Post-Cardiac Arrest Care: 2015 American Heart Association Guidelines Update for Cardiopulmonary Resuscitation and Emergency Cardiovascular Care. Circulation.

[16] Department of Statistics Singapore Population Trends. https://www.singstat.gov.sg/-/media/files/publications/population/population2020.pdf.

[17] Ong M.E., Shin S.D., Tanaka H., Ma M.H., Khruekarnchana P., Hisamuddin N., Atilla R., Middleton P., Kajino K., Leong B.S. (2011). Pan-Asian Resuscitation Outcomes Study (PAROS): Rationale, methodology, and implementation. Acad. Emerg. Med..

[18] Perkins G.D., Jacobs I.G., Nadkarni V.M., Berg R.A., Bhanji F., Biarent D., Bossaert L.L., Brett S.J., Chamberlain D., de Caen A.R. (2015). Cardiac arrest and cardiopulmonary resuscitation outcome reports: Update of the Utstein Resuscitation Registry Templates for Out-of-Hospital Cardiac Arrest: A statement for healthcare professionals from a task force of the International Liaison Committee on Resuscitation (American Heart Association, European Resuscitation Council, Australian and New Zealand Council on Resuscitation, Heart and Stroke Foundation of Canada, InterAmerican Heart Foundation, Resuscitation Council of Southern Africa, Resuscitation Council of Asia); and the American Heart Association Emergency Cardiovascular Care Committee and the Council on Cardiopulmonary, Critical Care, Perioperative and Resuscitation. Circulation.

[19] Loh J.P., Tan L.L., Zheng H., Lau Y.H., Chan S.P., Tan K.B., Chua T., Tan H.C., Foo D., Lee C.W. (2018). First Medical Contact-to-Device Time and Heart Failure Outcomes among Patients Undergoing Primary Percutaneous Coronary Intervention. Circ. Cardiovasc. Qual. Outcomes.

[20] Lim S.L., Smith K., Dyson K., Chan S.P., Earnest A., Nair R., Bernard S., Leong B.S., Arulanandam S., Ng Y.Y. (2020). Incidence and outcomes of out-of-hospital cardiac arrest in Singapore and Victoria: A collaborative study. J. Am. Heart Assoc..

[21] Geri G., Dumas F., Bougouin W., Varenne O., Daviaud F., Pène F., Lamhaut L., Chiche J.D., Spaulding C., Mira J.P. (2015). Immediate Percutaneous Coronary Intervention Is Associated with Improved Short- and Long-Term Survival after out-of-Hospital Cardiac Arrest. Circ. Cardiovasc. Interv..

[22] Jentzer J.C., Scutella M., Pike F., Fitzgibbon J., Krehel N.M., Kowalski L., Callaway C.W., Rittenberger J.C., Reynolds J.C., Barsness G.W. (2018). Early coronary angiography and percutaneous coronary intervention are associated with improved outcomes after out of hospital cardiac arrest. Resuscitation.

[23] Vyas A., Chan P.S., Cram P., Nallamothu B.K., McNally B., Girotra S. (2015). Early Coronary Angiography and Survival after out-of-Hospital Cardiac Arrest. Circ. Cardiovasc. Interv..

[24] Kim M.J., Ro Y.S., Shin S.D., Song K.J., Ahn K.O., Hong S.O., Kim Y.T. (2015). Association of emergent and elective percutaneous coronary intervention with neurological outcome and survival after out-of-hospital cardiac arrest in patients with and without a history of heart disease. Resuscitation.

[25] Sideris G., Voicu S., Yannopoulos D., Dillinger J.G., Adjedj J., Deye N., Gueye P., Manzo-Silberman S., Malissin I., Logeart D. (2014). Favourable 5-year postdischarge survival of comatose patients resuscitated from out-of-hospital cardiac arrest, managed with immediate coronary angiogram on admission. Eur. Heart J. Acute Cardiovasc. Care.

[26] Cronier P., Vignon P., Bouferrache K., Aegerter P., Charron C., Templier F., Castro S., El Mahmoud R., Lory C., Pichon N. (2011). Impact of routine percutaneous coronary intervention after out-of-hospital cardiac arrest due to ventricular fibrillation. Crit Care.

[27] Wijesekera V.A., Mullany D.V., Tjahjadi C.A., Walters D.L. (2014). Routine angiography in survivors of out of hospital cardiac arrest with return of spontaneous circulation: A single site registry. BMC Cardiovasc. Disord..

[28] Kern K.B., Lotun K., Patel N., Mooney M.R., Hollenbeck R.D., McPherson J.A., McMullan P.W., Unger B., Hsu C.H., Seder D.B. (2015). Outcomes of Comatose Cardiac Arrest Survivors with and without ST-Segment Elevation Myocardial Infarction: Importance of Coronary Angiography. JACC Cardiovasc. Interv..

[29] Neumar R.W., Nolan J.P., Adrie C., Aibiki M., Berg R.A., Böttiger B.W., Callaway C., Clark R.S., Geocadin R.G., Jauch E.C. (2008). Post-cardiac arrest syndrome: Epidemiology, pathophysiology, treatment, and prognostication. A consensus statement from the International Liaison Committee on Resuscitation (American Heart Association, Australian and New Zealand Council on Resuscitation, European Resuscitation Council, Heart and Stroke Foundation of Canada, InterAmerican Heart Foundation, Resuscitation Council of Asia, and the Resuscitation Council of Southern Africa); the American Heart Association Emergency Cardiovascular Care Committee; the Council on Cardiovascular Surgery and Anesthesia; the Council on Cardiopulmonary, Perioperative, and Critical Care; the Council on Clinical Cardiology; and the Stroke Council. Circulation.

[30] O′Connor R.E., Bossaert L., Arntz H.R., Brooks S.C., Diercks D., Feitosa-Filho G., Nolan J.P., Vanden Hoek T.L., Walters D.L., Wong A. (2010). Acute Coronary Syndrome Chapter Collaborators. Part 9: Acute coronary syndromes: 2010 International Consensus on Cardiopulmonary Resuscitation and Emergency Cardiovascular Care Science with Treatment Recommendations. Circulation.

[31] Zipes D.P., Camm A.J., Borggrefe M., Buxton A.E., Chaitman B., Fromer M., Gregoratos G., Klein G., Moss A.J., Myerburg R.J. (2006). ACC/AHA/ESC 2006 Guidelines for Management of Patients with Ventricular Arrhythmias and the Prevention of Sudden Cardiac Death: A report of the American College of Cardiology/American Heart Association Task Force and the European Society of Cardiology Committee for Practice Guidelines (writing committee to develop Guidelines for Management of Patients with Ventricular Arrhythmias and the Prevention of Sudden Cardiac Death): Developed in collaboration with the European Heart Rhythm Association and the Heart Rhythm Society. Circulation.

[32] Dankiewicz J., Nielsen N., Annborn M., Cronberg T., Erlinge D., Gasche Y., Hassager C., Kjaergaard J., Pellis T., Friberg H. (2015). Survival in patients without acute ST elevation after cardiac arrest and association with early coronary angiography: A post hoc analysis from the TTM trial. Intensive Care Med..

[33] Baldi E., Schnaubelt S., Caputo M.L., Klersy C., Clodi C., Bruno J., Compagnoni S., Benvenuti C., Domanovits H., Burkart R. (2021). Association of Timing of Electrocardiogram Acquisition after Return of Spontaneous Circulation with Coronary Angiography Findings in Patients with out-of-Hospital Cardiac Arrest. JAMA Netw. Open.

