# Supplementary material for: Early Coronary Angiography Is Associated with Improved 30-Day Outcomes among Patients with Out-of-Hospital Cardiac Arrest
Source: J Clin Med. 2021 Nov 6;10(21):5191. doi: 10.3390/jcm10215191 (PMC8584598; doi:10.3390/jcm10215191)
Supplement: Supplementary file 1 [file jcm-10-05191-s001.zip › jcm-1450832-supplementary.pdf]

Table S1: Summary of TTM practices according to institution

	Inclusion Criteria	Mode of TTM	Target Temperature	Duration of TTM
Institution A	Initial shockable rhythm	Induction with cold saline, followed by:<br>(1) Intravascular cooling (ZOLL Thermogard XP®), or<br>(2) Surface cooling (Blanketrol II or Cincinnati Sub-Zero,Arctic Sun™)	34°C	24 hours
Institution B	Initial shockable rhythm<br>Time to ROSC < 30 mins	Induction with cold saline, followed by:<br>Surface cooling (Gaymar Meditherm III)	32-34 ^{o}C	24 hours
Institution C	All rhythms	Surface cooling (Blanketrol II)	33 ^{o}C	24 hours
Institution D	All rhythms	Surface cooling (Cincinnati Sub-Zero,Arctic Sun™)	33 ^{o}C	24 hours
Institution E	All rhythms<br>Time to ROSC < 30 mins	Surface cooling (Blanketrol II)<br>Supplementation with cold saline if necessary	32-34 ^{o}C	12-24 hours

Table S1 summarizes the TTM practices according to institution. Protocols for 5 out of 6 institutions were available.

Abbreviations; TTM, targeted temperature management; ROSC, return of spontaneous circulation.

Table S2: Logistic regression of predictors for early CAG

	Early CAG	
**Variable**	**Adjusted OR (95% CI)**	**P-value**
Age	0.99(0.97,1.00)	0.016
Male sex	2.13(1.47,3.13)	<0.001
Heart disease	0.65(0.48,0.88)	0.006
Arrest at home	0.64(0.48,0.87)	0.004
Witnessed arrest	0.94(0.64,1.36)	0.732
Initial shockable rhythm	3.41(2.50,4.66)	<0.001
Bystander CPR	0.99(0.72,1.36)	0.941
CA to CPR >4 min	0.52(0.33,0.80)	0.003
Epinephrine administration	1.27(0.94,1.71)	0.115

Abbreviations: CAG, coronary angiography; OR, odds ratio; CPR, cardiopulmonary resuscitation; CA to CPR, time from cardiac arrest to first cardiopulmonary resuscitation.

Table S3: Characteristics of resuscitated adult OHCA patients who received CAG

	With PCI (n=277)	Without PCI (n=124)	P value*
**Demographics**			
Mean age (yrs), mean(SD)	59.3(11.1)	61.5(13.1)	0.077
Male sex, n (%)	240(86.6)	99(79.8)	0.082
Race			0.243
- Chinese, n(%)	164 (59.2)	85 (68.5)	
- Malay, n(%)	41 (14.8)	15 (12.1)	
- Indian, n(%)	53 (19.1)	15 (12.1)	
- Others, n	19 (6.9)	9 (7.3)	
**Medical History**			
Hypertension, n(%)	141(50.9)	76(61.3)	0.054
Diabetes mellitus, n(%)	81(29.2)	32(25.8)	0.480
Hyperlipidaemia, n(%)	106(38.3)	55(44.4)	0.250
Heart disease, n(%)	97(35.0)	71(57.3)	<0.001
Renal disease, n(%)	23(8.3)	18(14.5)	0.058
Respiratory disease, n(%)	17(6.1)	6(4.8)	0.605
Stroke, n(%)	15(5.4)	9(7.3)	0.472
**Event information**			
Arrest at home residence, n(%)	121(43.7)	59(47.6)	0.468
Witnessed arrest, n(%)	232(83.8)	97(78.2)	0.182
Initial shockable rhythm, n(%)**	200(72.2)	75(60.5)	0.019

Numbers are n (%) for categorical variables and mean(SD) for age. P-value is for differences between CAG with PCI versus CAG without PCI by X_2 test for categorical variables and Wilcoxon sum rank test for continuous variables.

Abbreviations: OHCA, out-of-hospital cardiac arrest; CAG, coronary angiography; PCI; percutaneous coronary intervention.

* Statistically significant at 5%.
** Initial shockable rhythm refers to first arrest rhythm which was ventricular fibrillation, ventricular tachycardia or unknown shockable rhythm

Table S4: Association between early CAG with outcomes

(a): Early CAG regardless of revascularization

		**30-day survival**		**Discharged with CPC 1 or 2**	
	**Variable**	**OR (95% CI)**	**P-value**	**OR (95% CI)**	**P-value**
**Unadjusted**	**Early CAG**	3.04(2.29,4.05)	<0.001	2.99(2.17,4.13)	<0.001
**Adjusted (Logistic regression)**	**Early CAG**	1.59(1.12,2.26)	0.010	1.54(1.01,2.34)	0.045
**Adjusted (IPWE)**	**Early CAG**	1.34(0.97,1.84)	0.073	1.26(0.88,1.80)	0.202

(b): Early CAG with and without immediate PCI

		**30-day survival**		**Discharged with CPC 1 or 2**	
	**Variable**	**OR (95% CI)**	**P-value**	**OR (95% CI)**	**P-value**
**Unadjusted**	**No/Delayed CAG**	Reference		Reference	
	**Early CAG without PCI**	1.64(1.04,2.58)	0.032	1.61(0.95,2.73)	0.080

	**Early CAG and immediate PCI**	3.95(2.87,5.43)	<0.001	3.78(2.67,5.36)	<0.001
**Adjusted (Logistic regression)**	**No/Delayed CAG**	Reference		Reference	
	**Early CAG without PCI**	1.06(0.62,1.80)	0.843	0.99(0.52,1.91)	0.984
	**Early CAG and immediate PCI**	1.91(1.29,2.84)	0.001	1.82(1.15,2.88)	0.010
**Adjusted (IPWE)**	**No/Delayed CAG**	Reference		Reference	
	**Early CAG without PCI**	0.83(0.49,1.39)	0.472	0.66(0.36,1.19)	0.165
	**Early CAG and immediate PCI**	1.71(1.19,2.44)	0.004	1.67(1.14,2.47)	0.009

(c): Early CAG with and without immediate PCI (pre-hospital defibrillation included)

		30-day survival		Discharged with CPC 1 or 2	
	**Variable**	**OR (95% CI)**	**P-value**	**OR (95% CI)**	**P-value**
**Unadjusted**	**No/Delayed CAG**	Reference		Reference	
	**Early CAG without PCI**	1.64(1.04,2.58)	0.032	1.61(0.95,2.73)	0.080
	**Early CAG and immediate PCI**	3.95(2.87,5.43)	<0.001	3.78(2.67,5.36)	<0.001
**Adjusted (Logistic regression)**	**No/Delayed CAG**	Reference		Reference	
	**Early CAG without PCI**	1.04(0.61,1.77)	0.891	0.99(0.52,1.91)	0.983

	**Early CAG and immediate PCI**	1.86 (1.25,2.76)	0.002	1.82(1.15,2.88)	0.011
**Adjusted (IPWE)**	**No/Delayed CAG**	Reference		Reference	
	**Early CAG without PCI**	0.84(0.49,1.45)	0.528	0.65(0.36,1.20)	0.168
	**Early CAG and immediate PCI**	1.61(1.12,2.32)	0.011	1.60(1.08,2.37)	0.020

Association between early CAG and outcomes using the categorical variable defined by (a) no or delayed CAG (reference) versus early CAG, (b) no or early CAG (reference) versus early CAG without PCI versus early CAG with immediate PCI, and (c) no or early CAG (reference) versus early CAG without PCI versus early CAG with immediate PCI, with pre-hospital defibrillation included in the model. Results were based on analyses performed using imputed dataset. Findings were similar to that without imputation.

Abbreviations: CAG, coronary angiography; CPC, Cerebral Performance Category; OR, odds ratio; CI, confidence interval; IPWE, inverse probability weighted estimator; PCI, percutaneous coronary intervention;

Figure S1: Distributional balance for propensity score

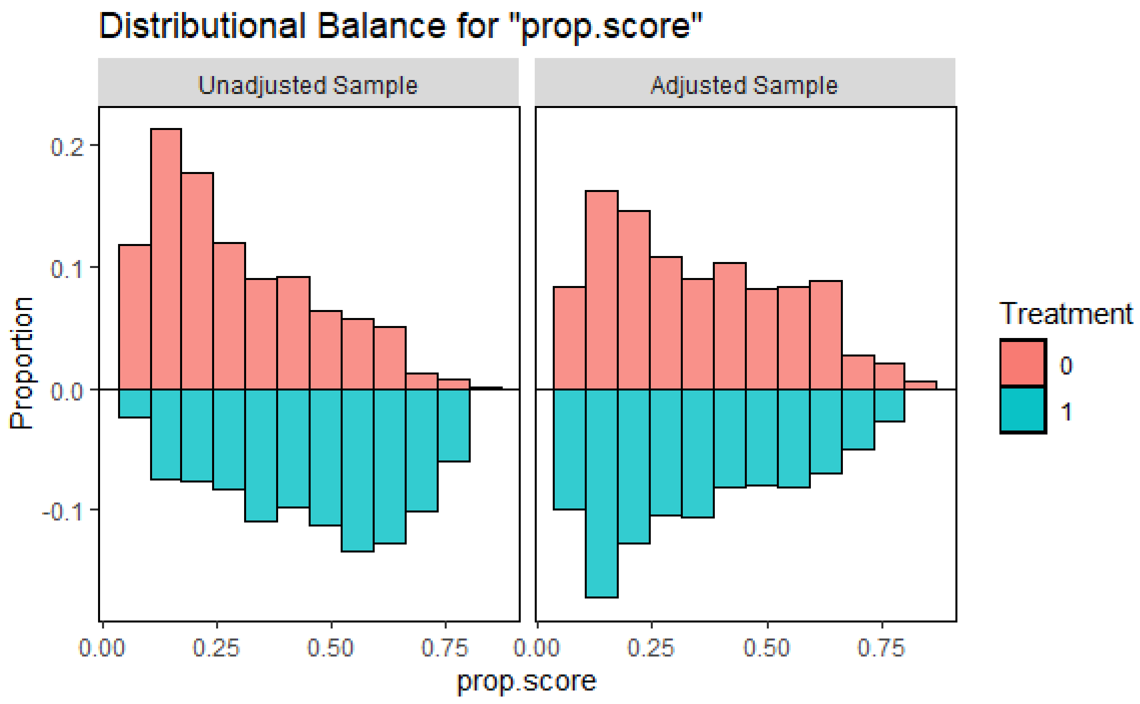

Figure S2: Covariate balance before and after IPWE

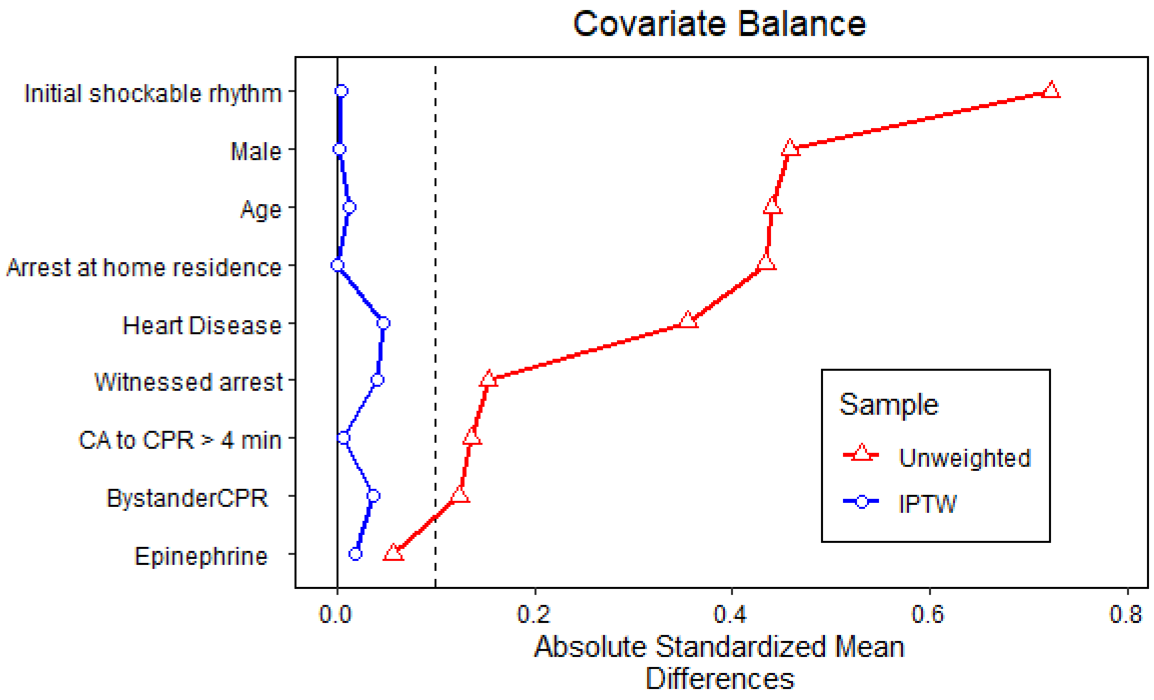

Abbreviations: IPWE, inverse probability weighted estimator; CA to CPR, time from cardiac arrest to first cardiopulmonary resuscitation; CPR, cardiopulmonary resuscitation; IPTW, inverse probability treatment weighting.
